# Sinonasal Inverted Papilloma and Squamous Cell Carcinoma: Contemporary Management and Patient Outcomes

**DOI:** 10.3390/cancers14092195

**Published:** 2022-04-28

**Authors:** Jacob G. Eide, Kevin C. Welch, Nithin D. Adappa, James N. Palmer, Charles C. L. Tong

**Affiliations:** 1 Department of Otorhinolaryngology-Head and Neck Surgery, Perelman School of Medicine, University of Pennsylvania, Philadelphia, PA 19104, USA; jacob.eide@pennmedicine.upenn.edu (J.G.E.); nithin.adappa@pennmedicine.upenn.edu (N.D.A.); james.palmer@pennmedicine.upenn.edu (J.N.P.); 2 Department of Otolaryngology-Head and Neck Surgery, Feinberg School of Medicine, Northwestern University, Chicago, IL 60611, USA; kevin.welch@nm.org

**Keywords:** Schneiderian, papilloma, tumor, recurrence, endoscopic, open, sinonasal

## Abstract

**Simple Summary:**

Inverted papillomas are benign sinonasal tumors that can recur or become cancerous. The mainstay of treatment is surgical resection. We summarize the biology of inverted papillomas and review surgical outcomes in an effort to define the current treatment strategy.

**Abstract:**

Inverted papillomas (IP) are the most common sinonasal tumor with a tendency for recurrence, potential attachment to the orbit and skull base, and risk of malignant degeneration into squamous cell carcinoma (SCC). While the overall rate of recurrence has decreased with the widespread adoption of high-definition endoscopic optics and advanced surgical tools, there remain challenges in managing tumors that are multiply recurrent or involve vital neurovascular structures. Here, we review the state-of-the-art diagnostic tools for IP and IP-degenerated SCC, contemporary surgical management, and propose a surveillance protocol.

## 1. Background

**Figure 1:**
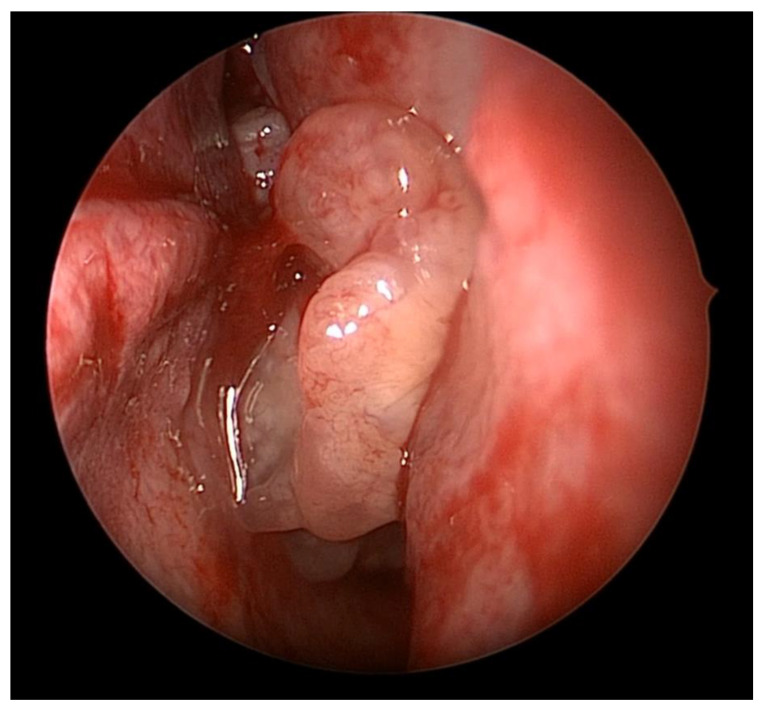
Biopsy-proven sinonasal inverted papilloma pedicled on the lateral nasal wall.

Inverted papillomas (IP) are mostly benign sinonasal tumors that are characterized by local destruction, tendency for recurrence, and risk of malignant degeneration [1]. The World Health Organization has defined three subtypes of sinonasal Schneiderian papillomas: inverted, exophytic, and oncocytic lesions [2]. Inverted papillomas (IPs) represent the most common subset of Schneiderian sinonasal papillomas, accounting for 62% of cases overall [2]. The tumor occurs with an incidence of 0.2 to 1.5/100,000 per year, is more frequent in males, and has a mean age of diagnosis of 55 years [1]. IPs can be a diagnostic challenge given that they often present with symptoms similar to an inflammatory process (e.g., rhinorrhea, nasal obstruction) and can be mistaken for a polyp, although these symptoms tend to be more unilateral when compared with allergic or inflammatory diseases. The pathogenesis of these tumors has not been clearly elucidated, but there is some evidence that suggests a potential role of inflammation as some IPs are associated with contralateral inflammation independent of the tumor on CT imaging [3,4]. As a result, the patient may have bilateral symptoms despite having an IP. This adds a diagnostic challenge as the presence of bilateral obstructive symptoms alone should not be used to rule out IP. Clinically, IPs can be distinguished from inflammatory polyps as they appear to be firm, non-translucent, and tend to have a poly-lobulated appearance (Figure 1).

While the exact etiology of IP remains unknown, environmental exposures have been proposed and a potential role for human papilloma virus (HPV) has been suspected since the 1980s. Studies have found HPV more frequently in tumors with dysplasia or malignant degeneration—specifically the HPV 16 and 18 serotypes [5]. Two recent meta-analyses suggested an association between HPV infection in IP tissue with progression to malignancy (pooled odds ratio 1.80–2.38), primarily from high-risk HPV subtypes 16 and 18 (pooled log OR 1.67 and 2.68, respectively) [6,7]. Interestingly, more recent studies have found that *EGFR* mutations are present in the majority of IPs (72–90%) and a significant portion of squamous cell carcinoma arising from IP (30–88%) [8,9,10]. When inverted and oncocytic papillomas were tested, HPV DNA was only found in a limited number of patients (6.1% of IPs and 11.1% of oncocytic papillomas) [11]. Similarly, p16 expression, which has been used as a surrogate for HPV-driven oncogenesis, was not found in most samples (22.4% IP, 27.8% oncocytic papilloma) [11]. It has thus been suggested that both HPV and *EGFR* mutations can cause IP transformation to SCC and represent distinct pathways to tumorigenesis [12]. Overall, the incidence of identifying squamous cell carcinoma at the time of initial resection is approximately 5–11%, with a 3.6–18% incidence of developing malignancy at the time of recurrence [13,14,15,16]. It has been estimated that having a diagnosis of IP increases the risk of SCC by 3–10% [13]. It is not unusual for a tumor to harbor varying degrees of dysplasia with only focal invasive disease. As such, the mainstay of IP treatment is complete extirpation that allows pathologic examination and staging, with adjuvant therapy reserved for unresectable or invasive disease.

## 2. Preoperative Evaluation

Initial evaluation of patients suspected to have an intranasal mass begins with a comprehensive history and physical examination, including a nasal endoscopy. History should specifically address previous history of nasal mass or polyp removals, tobacco use or exposure, and symptoms related to mass effect (nasolacrimal obstruction, vision changes, headaches). Unilateral symptoms, negative allergic workup, and failure to respond to medical therapy for inflammatory processes should raise suspicion for sinonasal mass but are not specific to IP. Complete head and neck examination should be performed with attention to any visual deficits, numbness in the trigeminal nerve distribution, extraocular movement deficits, middle ear effusion, and presence of any cervical lymphadenopathy.

Classically, IPs are lobulated, firm tumors [1]. Symptoms are typically non-specific, including nasal obstruction, epistaxis, rhinorrhea, and facial pressure, and accordingly, IPs are commonly diagnosed in a delayed fashion [17]. Interestingly, in some series, as many as 23% of patients were asymptomatic from their mass, which was found incidentally [18]. Tissue confirmation is recommended prior to definitive resection and can frequently be accomplished in the clinic setting. However, IPs can be found concurrently with inflammatory polyps and the diagnosis should still be suspected even in cases of negative biopsy, as there has been a reported 17% false negative rate [1]. There are also data suggesting a higher degree of radiographic contralateral sinus mucosal thickening in IP cases compared to controls (58.9% vs. 26.7%), which has caused some investigators to consider the role of chronic inflammation in IP development [3].

## 3. Imaging

**Figure 2:**
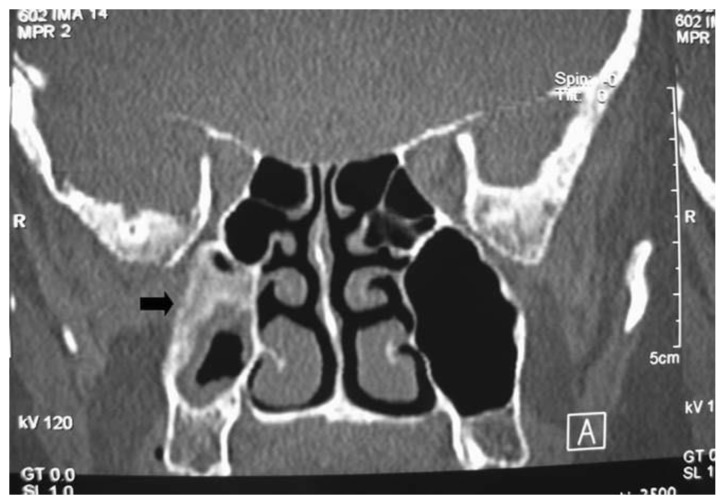
Coronal image of computed tomography showing a recurrent sinonasal inverted papilloma pedicled on the posterior maxillary sinus wall. There is significant hyperostosis at the origin of the lesion.

Preoperative imaging is routinely obtained to evaluate the extent of the tumor and assist in surgical planning. Non-contrast computed tomography (CT) scans with thin cuts (<1 mm) are standard protocol to evaluate for areas of bony erosion, although findings typically demonstrate a non-specific soft tissue density with microcalcifications present in 20% of cases [19]. CT is particularly sensitive at detecting areas of hyperostosis, which has been used to predict the site of IP attachment with positive predictive values of 89–95% reported [20,21,22]. (Figure 2) This finding on imaging could be corroborated with nasal endoscopy, but the point of insertion is often obscured during exam.

**Figure 3:**
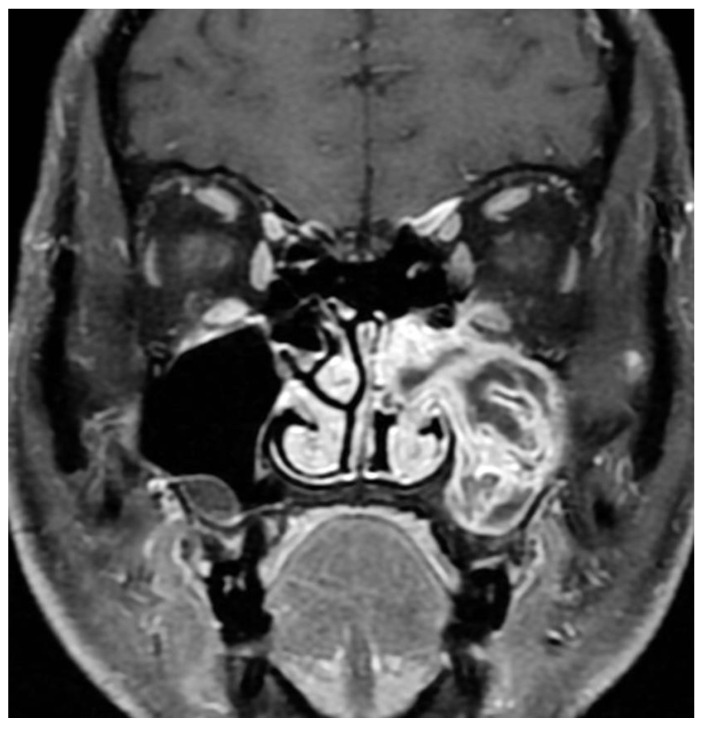
Alternating lines of high and low signal intensity (convoluted cerebriform pattern) seen in an inverted papilloma of the maxillary sinus.

Magnetic resonance imaging (MRI) has increasingly become part of preoperative workup due to IP’s unique imaging characteristics. Typical findings are a hypodensity on T1, iso- or hypodensity on T2, contrast enhancement, and convoluted cerebriform pattern (CCP) [23,24]. (Figure 3) This imaging modality is superior to CT in delineating the tumor from inspissated secretions and in predicting malignant degeneration [25]. Specifically, benign IPs have been found to have a higher prevalence of CCP compared to IP-transformed SCC, and a significantly lower apparent diffusion coefficient (ADC) on MRI diffusion-weighted imaging (DWI) [25]. One study with limited sample size suggested that MRI was better at predicting involvement of the frontal sinus than CT alone, with other studies suggesting that MRI is accurate at predicting the final pathologic stage of the IP in the vast majority of cases [26,27]. Frank bone erosion and aberration of the classic cerebriform appearance on MRI have been associated with malignant transformation, and some authors have suggested more advanced dynamic contrast MRI techniques as adjuncts [28].

## 4. Surgical Planning

**Figure 4:**
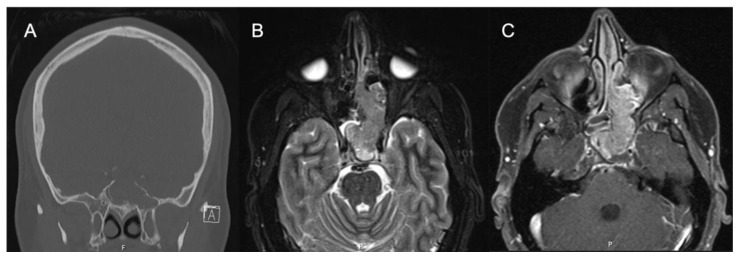
IP-degenerated squamous cell carcinoma. (**A**) Non-contrasted CT sinus demonstrating a mass of the left sphenoid with erosion of the skull base at the sella. (**B**) STIR sequence contrasted MRI demonstrating an expansile mass originating from the left sphenoid lateral wall. (**C**) T1 sequence contrasted MRI with fat suppression demonstrating a mass of the left sphenoid and ethmoid cavity.

Locally advanced IPs or IP-degenerated SCC require further workup and a collaborative approach. While most IPs can be managed by an experienced surgeon, rare tumors with significant extra-sinus extension or skull base involvement should prompt neurosurgical evaluation, and management should be undertaken by dedicated skull base teams for optimal results. Further workup to delineate involvement of key neurovascular structures may be required. Figure 4 shows images of a patient who was initially evaluated for headaches and confusion and was found to have a lesion of the sphenoid sinus with erosion of the sella and dehiscence of the carotid artery and optic nerve. Biopsy revealed the pathology as IP without dysplasia, and resection was performed with careful dissection off the neurovascular structures. 

**Figure 5:**
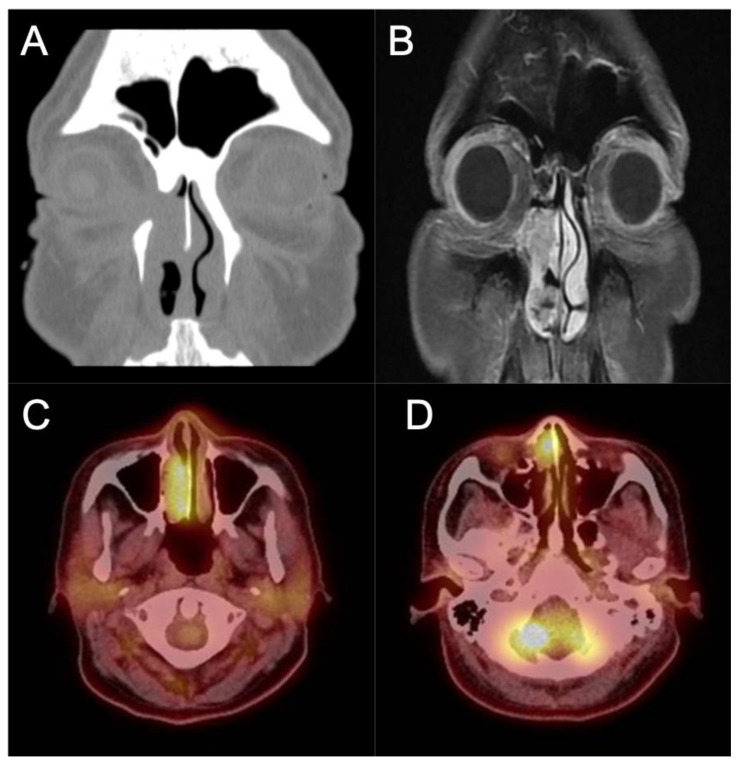
IP-degenerated squamous cell carcinoma. (**A**) Non-contrasted CT scan demonstrating an erosive mass in the right nasal cavity with invasion into the orbit. (**B**) T1 sequence contrasted MRI scan demonstrating the invasion of the mass into the medial orbit. (**C**,**D**) PET CT scan demonstrating avidity of the right nasal mass.

Involvement of the orbit should likewise prompt referral to ophthalmology evaluation for baseline exam and joint management. Figure 5 contains images of a patient who presented to our office with recurrent epiphora after an external dacryocystorhinostomy (DCR) over a year ago. The patient also noted chronic unilateral nasal obstruction and a history of minor procedures for polyp removal spanning two decades outside of the United States. An endoscopic exam revealed an intranasal mass which was biopsy positive for IP-degenerated SCC. The coronal CT cut shows the bony dehiscence that was likely a result of her prior DCR, and the T1-weighted contrast enhanced MRI image shows extension of the tumor into the orbit and infiltration into the periorbita. A PET/CT demonstrated local FDG avidity without regional or distant uptake. This patient subsequently underwent an endoscopic-assisted resection with a modified Denker’s procedure and lateral rhinotomy, resection of the periorbita, and reconstruction with canthoplasty.

## 5. Staging

**Figure 6:**
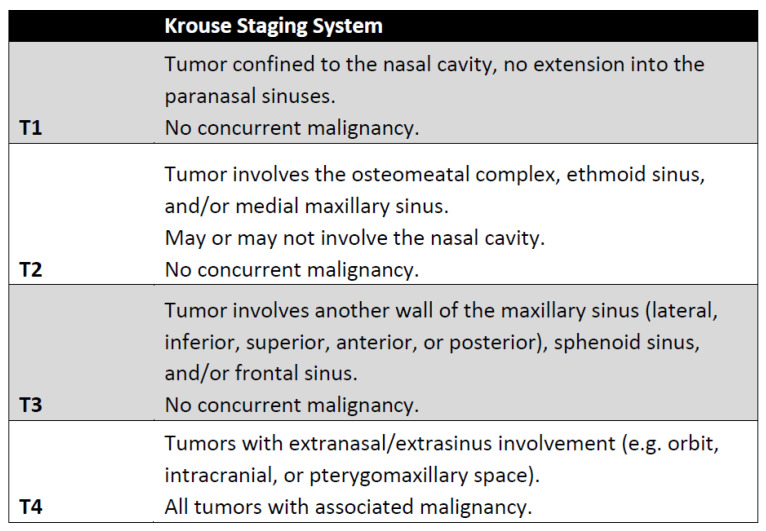
Krouse staging system [20].

There have been several proposed staging systems for IP, but perhaps the most commonly used is the Krouse staging system (Figure 6) [29]. It is important to note that this system was developed in the pre-endoscopic era and based on prior surgical experience and other staging systems for sinonasal malignancies. It also prioritized ease of use, and at the time was not stratified based on patient outcomes. Kim et al. performed a meta-analysis investigating recurrence based on surgical approach and completed a sub-group analysis of 4 papers that included the Krouse classification. They noted a higher risk ratio for recurrence in higher Krouse classifications but their data did not achieve significance [30]. Lisan et al. performed a subsequent meta-analysis of 13 studies, specifically designed to investigate the association with the Krouse system with recurrence. Interestingly, they found that there was no significant difference in recurrence between T1 and T2 lesions or between T3 and T4 lesions. There was, however, a 51% increase in recurrence between T2 and T3 lesions [31]. These findings highlight the fact that while the Krouse classification allows for standardization of reporting these tumors, there is a significant amount of heterogeneity within groups, particularly within the T4 group that included all carcinomas. A number of other staging systems have since been proposed, including the Oikawa, Han, Cannady, and Meng systems, but there is limited evidence that these systems correlate with tumor recurrence [32,33].

## 6. Pathology and Molecular Changes Associated with Malignant Degeneration

**Figure 7:**
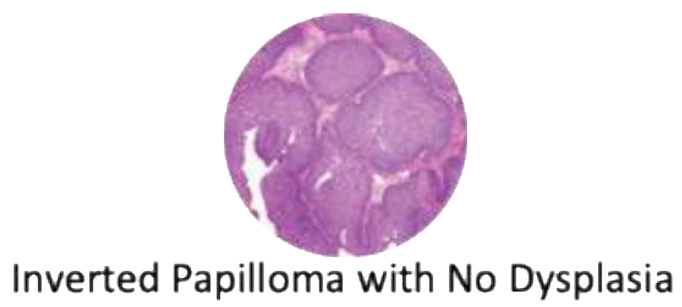
Hematoxylin and eosin staining of an inverted papilloma demonstrating ribbons of hyperplastic respiratory epithelium that grow into the adjacent stroma.

Histologically, IPs are characterized by a thickened epithelium enclosed by the basement membrane on hematoxylin and eosin stain, thus appearing “inverted” compared to other Schneiderian papillomas (Figure 7) [34]. Varying degrees of dysplasia can be observed in the non-keratinizing epithelium, ranging from none, mild, and moderate to severe dysplasia. The degree of dysplasia should be noted by the examining pathologist with attention to possible SCC or other concurrent malignancies [34]. Although HPV has long been implicated in the pathogenesis of IP, detection rate has not been consistent across techniques (PCR, DNA/mRNA in situ hybridization) and are not routinely performed. It has been reported, however, that high-risk HPV in IP is correlated with an increase in epidermal growth factor receptor (EGFR) expression, which leads to dysplasia and invasion [35]. There is also a growing interest in understanding the molecular pathways that lead to carcinogenesis. One area of interest is epigenetic changes that modify gene expression. In a comparative study between 15 patients with IP and 12 with SCC ex-IP, three genes were identified (*OPA3*, *MIR661*, and *PLEC)* at six different genetic sites that were hypermethylated compared to controls. *MIR661* encodes a microRNA that can either promote or suppress tumor aggressiveness depending on p53 expression, and miR-661 mRNA was significantly upregulated in SCC ex-IP [36,37]. The OPA3 protein is a key portion of the mitochondrial outer membrane and is found to be decreased in SCC ex-IP [36,38]. Plectin, the protein encoded by *PLEC*, regulates the intermediate filament structure of cells and was noted to be upregulated in SCC ex-IP, similar to reports in ovarian and pancreatic tumors [36,39,40].

MicroRNAs are small, non-coding RNA segments that regulate gene expression via messenger RNA cleavage and inhibition of translation to cell proteins [41]. They represent another level of transcriptional regulation that, when aberrant, can lead to cancer progression. Analysis of microRNA expression in SCC ex-IP compared to IP demonstrated a significantly higher level of miR-296-3p [42]. This microRNA subsequently downregulates PTEN, a known tumor suppressor via inhibition of the phosphoinositide 3-kinase (PI3K)/protein kinase B (Akt) pathway [42,43]. PTEN depletion has been reported to be associated with worse outcomes in non-sinonasal SCC and may be an important prognostic factor for SCC ex-IP in the future [44]. 

There have been multiple studies looking at gene expression and mutation in SCC ex-IP. A recent systematic review has summarized the results of various genomic sequencing of IP and SCC ex-IP, finding progressively increasing numbers of mutated genes as the samples became more dysplastic [45]. They specifically found that *KRAS*, *APC*, and *STK11* genes were mutated at a higher rate in SCC arising from IP [45]. Interestingly, other authors also noted somatic EGFR mutations in IP degenerated SCC and only found KRAS mutations in malignancies related to oncocytic papilloma, albeit in a limited number of patient samples [46]. Genomic sequencing of IP associated SCC has also noted mutations in lysine methyltransferase 2A (*KMT2D*), cyclin-dependent kinase inhibitor 2A (*CDKN2A*), tumor protein 53 (*TP53*), phosphodiesterase 4D interacting protein (*PDE4DIP*), and neurofibromin 1 (*NF1*) [47]. Parallel work has identified FoxM1 as a proliferation transcription factor that has been associated with a variety of malignancies and has been found to be significantly elevated in inverted papilloma and SCC ex-IP [48]. Importantly, expression of FoxM1 was found to correlate with Krouse stage and histological grade, suggesting that it plays a role in the transition from normal epithelium to IP and malignancy [48]. Finally, EGFR mutations have been identified in 88% of IPs and 77% of IP-SCC but not identified in de novo SCC. EGFR inhibitors have been shown to be effective in vitro and may be a viable treatment for IPs and IP-SCC in the future [8,49]. While the exact etiology of carcinogenesis remains unclear, recent genomic studies implicating specific molecular pathways appear to be promising. Table 1 summarizes the known molecular abnormalities suspected in IP malignant transformation.

## 7. Surgical Management and Outcomes 

### 7.1. Endoscopic versus Open Surgery for IP

Intuitively, complete surgical excision of IP lesions is essential to long-term disease control and prevention of recurrence. Prior to the mid-1990s, open en-bloc surgical resection was the standard-of-care with associated lateral rhinotomy or Caldwell–Luc approaches [50]. As instrumentation and optics have greatly advanced in the years that followed, endoscopic resection has gradually been adopted as the preferred approach [51]. Much of the interest in subsequent research focused on comparing the patient outcomes of endoscopic and open approaches. In a landmark study, Busquets and Hwang performed a meta-analysis and compared the historical pre-endoscopic cohort to contemporary patients treated via open surgery and found similar rates of recurrence (19% vs. 20%). However, when the contemporary endoscopic cohort was compared to open surgery, they found a lower rate of recurrence in the endoscopically treated patients (20% vs. 12%, respectively) [52]. Follow-up studies a decade later by Kim et al. confirmed a 44% (RR = 0.56) decrease in recurrence with endoscopic or endoscopic-assisted surgery, with Peng et al. showing lower recurrence in endoscopic cases (endoscopic alone 12.80%, open 16.58%, combined 12.60%) [30,53], Finally, another meta-analysis by Goudakos et al. showed similar findings (13.8% endoscopic, 18.7% open, and 12.9% combined) [54].

Currently, only few would question the endoscopic approach in achieving oncologic resection. Tumors that are limited in size and attached in anatomic areas accessible by the endoscope and instruments would often undergo endoscopic resection, with open or endoscopic-assisted approaches reserved for large and/or lateral frontal sinus involvement, anterolateral/inferior maxillary sinus lesions (although some suggest endoscopic medial maxillectomy is sufficient exposure), and cases of carcinoma or extra-sinus extension [17,55,56]. Ultimately, the decision on surgical approach is tailored to the patient’s extent of disease. The ability in achieving negative margins irrespective of technique is paramount to recurrence-free survival, while some support the use of intraoperative frozen sections [57].

### 7.2. Attachment-Oriented Surgery

With attachment-oriented surgery, the identification of the site of attachment is prioritized over traditional en-bloc resection. With the advent of high-definition optics, tissue debridement/suction systems, and high-speed drills, large volume tumors can now be debulked until the underlying bony attachment site is identified. This site can often be marked by an area of hyperostosis on CT scans and intraoperative navigation systems. The tumor release site should ideally be subperiosteal to remove pockets of tumor with drilling of the underlying bone to ensure complete resection [55]. Past studies have revealed tumor attachment to the underlying bone, highlighting the need for bony drilling after resection [58]. A minority of IP tumors have multiple attachment sites, either in the same anatomic site or multiple sites (20.5% two attachment sites, 1.9% three attachment sites), which increases rates of recurrence (OR 3.5) due to the aggressive nature of the disease and the increased surface area, and thus probability of a recurrence [59].

### 7.3. Surgical Techniques and Outcomes

As mentioned, the surgical approach to IP has changed dramatically, with early resections relying on open lateral rhinotomy and Weber–Ferguson incisions for access [60]. With improvement and experience with endoscopic techniques, the vast majority of IPs can be managed endoscopically. The most common site of origin for inverted papillomas is the lateral nasal wall, and the primary limitation to the endoscopic approach is the lateral extent of the tumor. Accordingly, endoscopic medial maxillectomies and modified endoscopic Denker’s procedures to access the far lateral maxillary sinus have been described [61]. A sublabial (Caldwell–Luc) approach also offers excellent lateral view. Finally, an endoscopic dacryocystorhinostomy (DCR) should be considered if there was gross involvement of the nasolacrimal duct or if the duct was transected during the approach to prevent postoperative epiphora.

**Figure 8:**
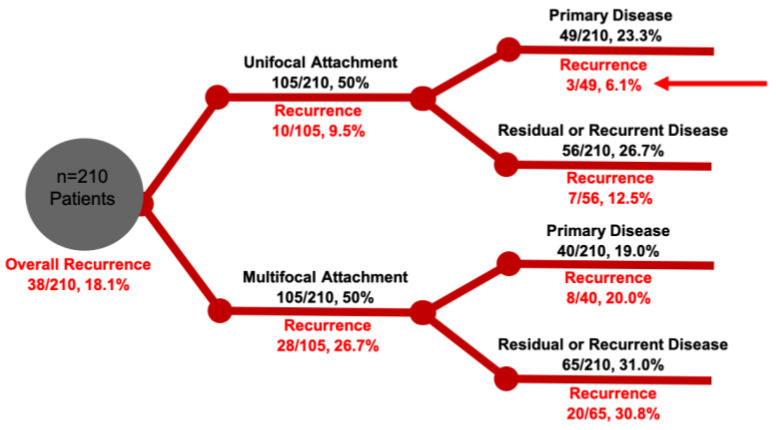
IP outcomes stratified by attachment (unifocal vs. multifocal), primary or residual/recurrent disease by a single institution. Red arrow denotes optimal outcome after management of unifocal primary disease.

A common criticism of studies comparing the surgical approaches has been patient selection bias. In a large contemporary cohort of a single tertiary rhinology practice, it became clear that disease severity (as determined by focality of the tumor attachment) and primary surgery (as opposed to revision surgery) have great implications in recurrence rates. In patients with only a solitary focus of attachment, the overall recurrence was found to be 9.5% (only 6.1% in those with primary resection, 12.5% in secondary cases), and 26.7% in multifocal IP (20% primary, 30.8% secondary) [59]. (Figure 8). These results reflect that multifocal IP reflect a more aggressive disease process that, despite primary surgery, these patients are at a higher risk for recurrence. It should also be noted that primary surgery confers a significant benefit in achieving a lower recurrence rate; thus, all efforts should be made to remove the entire disease at first attempt.

### 7.4. Management of IP-Associated Carcinoma In Situ

Similar to other epithelial lesions, IP can harbor mild, moderate, or severe dysplasia (carcinoma in situ/CIS). CIS represents the highest degree of dysplasia prior to invasive carcinoma and patients with CIS are considered at a higher risk of recurrence. In a retrospective review, 17.2% of IP resection specimens were noted to have CIS [62]. The patients who had CIS had a higher incidence of prior and current smoking (74.3% vs. 47.1% and 24.3% vs. 11.3%, respectively) [62]. Complete surgical excision of the lesion—including the IP and areas of CIS—is the preferred method of treatment with meticulous attention to treating the underlying bone to prevent leaving nests of cells that could lead to recurrence [63]. Although recurrence rates of IP associated CIS are relatively high at 27%, further degeneration into invasive SCC are rare at 2.7% [62]. Radiation therapy is typically reserved for tumors with invasive carcinoma, but it has been applied in patients with multiply recurrent dysplastic lesions at unresectable sites (typically the orbit and skull base) [62].

### 7.5. Management of IP-Degenerated Squamous Cell Carcinoma

Squamous cell carcinoma arising from IP is treated in a similar manner to primary sinonasal SCC with complete surgical resection followed by post-operative radiation therapy for higher stage tumors. As discussed above, the endoscopic technique is generally preferred unless there is extensive invasion of surrounding structures necessitating open approach. Retrospective reviews have reported an overall survival rate at 1, 3, and 5 years of 90.5%, 75.5%, and 68.5%, respectively [64]. Unsurprisingly, T4 disease and positive surgical margins are associated with worse survival [64]. For patients with sinonasal SCC, there is a survival benefit if the etiology of the SCC is from a transformed inverted papilloma when compared to de novo lesions. Early stage (Tis, T1, T2) SCC ex-IP appears to have a better survival than de novo SCC, but advanced disease (T3, T4) behaved similarly regardless of origin status [65]. As expected, distant metastasis is an independent predictor of poor prognosis but method of surgical resection (endoscopic vs. open) does not seem to impact survival rates [65]. This remains an active area of research and the possibility of potentially de-escalating therapy for early-stage SCC ex-IP is intriguing.

### 7.6. Post-Surgical Surveillance

Clinic visits with nasal endoscopy is often cost effective and key to early detection of recurrence. Surveillance schedules are variable, but exams at regular intervals for 3–5 years are consistent with most head and neck tumors, with some advocating lifetime follow-up due to the tumor’s propensity of delayed recurrences. It should be noted that the majority of recurrences occur at the primary site within the first two years of treatment, which are often felt to be residual disease rather than true recurrences [1]. There is significant evidence for late recurrence, with studies noting 26.1% of all recurrences occurred over three years from initial diagnosis, and rare recurrences up to 15 years after primary surgery. Post-operative imaging is often advocated for malignant disease, recurrent disease, or disease pedicled in challenging anatomical areas (e.g., optico-carotid recess, lateral wall of the frontal sinus).

## 8. Adjuvant Therapy 

The most common form of adjuvant therapy is external beam radiation therapy (XRT), which is typically reserved for SCC ex-IP following complete resection. Additionally, XRT should be considered for inoperable tumors (poor patient candidate, involvement of neurovascular structures) [1,13]. Patients should be counselled that for SCC ex-IP, complete surgical resection followed by XRT has a superior 5-year survival (84%) compared to 41% for XRT alone [66,67]. There has also been limited evidence of response to carboplatin/paclitaxel for inoperable IP with CIS that significantly debulked the tumor and allowed for surgical resection [68]. Finally, topical 5-fluorouracial has had positive preliminary results in recurrent challenging cases, although further investigation is needed [69].

## 9. Treatment Algorithm

**Figure 9:**
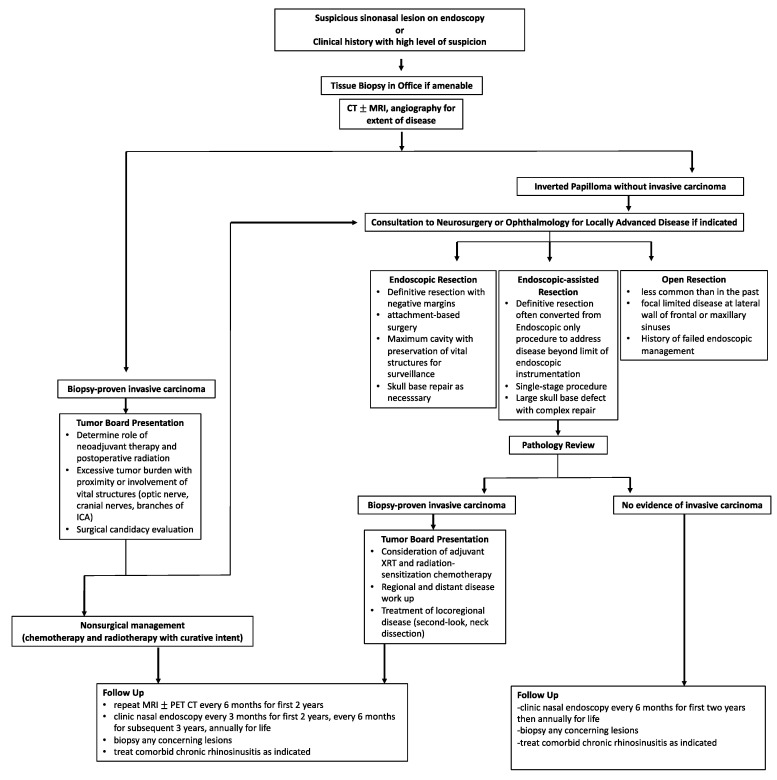
Treatment algorithm for the diagnosis, management, and surveillance of both IP and malignant SCC ex-IP.

Figure 9 summarizes the overall workup, management, and surveillance strategy for IPs and SCC ex-IP. The algorithm highlights the need for a multi-disciplinary approach for locally advanced tumors, proper patient counseling, and shared decision making in treatment modality and surveillance.

## 10. Conclusions and Future Directions 

As IP is the most common sinonasal tumor with a potential of malignant transformation, it will likely remain an active area of research for years to come. It offers a unique model to study sinonasal SCC, which despite moderate advances in surgical techniques and adjuvant therapy, the overall survival rate remains low. Our lack of understanding is highlighted by the fact that, to this day, IPs can only be confirmed histologically by H&E stain, without a validated set of molecular markers unique to the pathology. Recent genomic studies have shown promise in better classifying these tumors, with several signaling pathways implicated in the pathogenesis of IP and SCC ex-IP. The validation studies and in vitro inhibition experiments that follow should greatly enhance our understanding of their tumor biology and, ultimately, conceive novel treatment options for patients.

## Figures and Tables

**Table 1:** Summary of molecular changes implicated in malignant degeneration.

Molecular Change
***Hypermethylated Genes***
OPA3
MIR661
PLEC
***MicroRNA***
miR-296-3p
***Transcription Factor***
FoxM1
***Gene Mutations***
KRAS
APC
STK11
EGFR
KMT2D
CDKN2A
TP53
PDE4DIP
NF1

## References

[1] Lisan Q., Laccourreye O., Bonfils P. (2016). Sinonasal inverted papilloma: From diagnosis to treatment. Eur. Ann. Otorhinolaryngol. Head Neck Dis..

[2] Thompson L. (2006). World Health Organization classification of tumours: Pathology and genetics of head and neck tumours. Ear Nose Throat J..

[3] Papagiannopoulos P., Tong C.L., Kuan E.C., Tajudeen B.A., Yver C.M., Kohanski M.A., Cohen N.A., Kennedy D.W., Palmer J.N., Adappa N.D. (2020). Inverted papilloma is associated with greater radiographic inflammatory disease than other sinonasal malignancy. Int. Forum Allergy Rhinol..

[4] Orlandi R.R., Rubin A., Terrell J.E., Anzai Y., Bugdaj M., Lanza D.C. (2002). Sinus inflammation associated with contralateral inverted papilloma. Am. J. Rhinol..

[5] Lawson W., Schlecht N.F., Brandwein-Gensler M. (2008). The role of the human papillomavirus in the pathogenesis of Schneiderian inverted papillomas: An analytic overview of the evidence. Head Neck Pathol..

[6] Stepp W.H., Farzal Z., Kimple A.J., Ebert C.S., Senior B.A., Zanation A.M., Thorp B.D. (2021). HPV in the malignant transformation of sinonasal inverted papillomas: A meta-analysis. Int. Forum Allergy Rhinol..

[7] McCormick J.P., Suh J.D., Lee J.T., Wells C., Wang M.B. (2021). Role of High-Risk HPV Detected by PCR in Malignant Sinonasal Inverted Papilloma: A Meta-Analysis. Laryngoscope.

[8] Udager A.M., Rolland D.C.M., McHugh J.B., Betz B.L., Murga-Zamalloa C., Carey T.E., Marentette L.J., Hermsen M.A., DuRoss K.E., Lim M.S. (2015). High-Frequency Targetable EGFR Mutations in Sinonasal Squamous Cell Carcinomas Arising from Inverted Sinonasal Papilloma. Cancer Res..

[9] Sasaki E., Nishikawa D., Hanai N., Hasegawa Y., Yatabe Y. (2018). Sinonasal squamous cell carcinoma and EGFR mutations: A molecular footprint of a benign lesion. Histopathology.

[10] Sahnane N., Ottini G., Turri-Zanoni M., Furlan D., Battaglia P., Karligkiotis A., Albeni C., Cerutti R., Mura E., Chiaravalli A.M. (2019). Comprehensive analysis of HPV infection, EGFR exon 20 mutations and LINE1 hypomethylation as risk factors for malignant transformation of sinonasal-inverted papilloma to squamous cell carcinoma. Int. J. Cancer.

[11] Wang H., Zhai C., Liu J., Wang J., Sun X., Hu L., Wang D. (2020). Low prevalence of human papillomavirus infection in sinonasal inverted papilloma and oncocytic papilloma. Virchows Arch..

[12] Udager A.M., McHugh J.B., Goudsmit C.M., Weigelin H.C., Lim M.S., Elenitoba-Johnson K.S.J., Betz B.L., Carey T.E., Brown N.A. (2018). Human papillomavirus (HPV) and somatic EGFR mutations are essential, mutually exclusive oncogenic mechanisms for inverted sinonasal papillomas and associated sinonasal squamous cell carcinomas. Ann. Oncol..

[13] Mirza S., Bradley P.J., Acharya A., Stacey M., Jones N.S. (2007). Sinonasal inverted papillomas: Recurrence, and synchronous and metachronous malignancy. J. Laryngol. Otol..

[14] Lesperance M.M., Esclamado R.M. (1995). Squamous cell carcinoma arising in inverted papilloma. Laryngoscope.

[15] Karligkiotis A., Lepera D., Volpi L., Turri-Zanoni M., Battaglia P., Lombardi D., Accorona R., Bignami M., Nicolai P., Castelnuovo P. (2016). Survival outcomes after endoscopic resection for sinonasal squamous cell carcinoma arising on inverted papilloma. Head Neck.

[16] Anari S., Carrie S. (2010). Sinonasal inverted papilloma: Narrative review. J. Laryngol. Otol..

[17] Klimek T., Atai E., Schubert M., Glanz H. (2000). Inverted papilloma of the nasal cavity and paranasal sinuses: Clinical data, surgical strategy and recurrence rates. Acta Oto-Laryngol..

[18] Minovi A., Kollert M., Draf W., Bockmühl U. (2006). Inverted papilloma: Feasibility of endonasal surgery and long-term results of 87 cases. Rhinology.

[19] Momeni A.K., Roberts C.C., Chew F.S. (2007). Imaging of chronic and exotic sinonasal disease: Review. AJR Am. J. Roentgenol..

[20] Yousuf K., Wright E.D. (2007). Site of attachment of inverted papilloma predicted by CT findings of osteitis. Am. J. Rhinol..

[21] Lee D.K., Chung S.K., Dhong H.J., Kim H.Y., Kim H.J., Bok K.H. (2007). Focal hyperostosis on CT of sinonasal inverted papilloma as a predictor of tumor origin. AJNR Am. J. Neuroradiol..

[22] Bhalla R.K., Wright E.D. (2009). Predicting the site of attachment of sinonasal inverted papilloma. Rhinology.

[23] Savy L., Lloyd G., Lund V.J., Howard D. (2000). Optimum imaging for inverted papilloma. J. Laryngol. Otol..

[24] Jeon T.Y., Kim H.J., Chung S.K., Dhong H.J., Kim H.Y., Yim Y.J., Kim S.T., Jeon P., Kim K.H. (2008). Sinonasal inverted papilloma: Value of convoluted cerebriform pattern on MR imaging. AJNR Am. J. Neuroradiol..

[25] Yan C.H., Tong C.C.L., Penta M., Patel V.S., Palmer J.N., Adappa N.D., Nayak J.V., Hwang P.H., Patel Z.M. (2019). Imaging predictors for malignant transformation of inverted papilloma. Laryngoscope.

[26] Oikawa K., Furuta Y., Oridate N., Nagahashi T., Homma A., Ryu T., Fukuda S. (2003). Preoperative staging of sinonasal inverted papilloma by magnetic resonance imaging. Laryngoscope.

[27] Kasbekar A.V., Swords C., Attlmayr B., Kulkarni T., Swift A.C. (2018). Sinonasal papilloma: What influences the decision to request a magnetic resonance imaging scan?. J. Laryngol. Otol..

[28] Zhang L., Fang G., Yu W., Yang B., Wang C., Zhang L. (2020). Prediction of malignant sinonasal inverted papilloma transformation by preoperative computed tomography and magnetic resonance imaging. Rhinology.

[29] Krouse J.H. (2000). Development of a staging system for inverted papilloma. Laryngoscope.

[30] Kim J.S., Kwon S.H. (2017). Recurrence of sinonasal inverted papilloma following surgical approach: A meta-analysis. Laryngoscope.

[31] Lisan Q., Moya-Plana A., Bonfils P. (2017). Association of Krouse Classification for Sinonasal Inverted Papilloma With Recurrence: A Systematic Review and Meta-analysis. JAMA Otolaryngol.-Head Neck Surg..

[32] Nakayama T., Tsunemi Y., Kashiwagi T., Kuboki A., Yamakawa S., Konno W., Mori A., Iimura J., Tsukidate T., Tanaka Y. (2021). Comparison of Current Staging Systems for Sinonasal Inverted Papilloma. Am. J. Rhinol. Allergy.

[33] Mak W., Webb D., Al-Salihi S., Dadgostar A., Javer A. (2018). Sinonasal inverted papilloma recurrence rates and evaluation of current staging systems. Rhinology.

[34] Centre International de Recherche sur le Cancer (2005). Pathology and Genetics of Head and Neck Tumours.

[35] Re M., Gioacchini F.M., Bajraktari A., Tomasetti M., Kaleci S., Rubini C., Bertini A., Magliulo G., Pasquini E. (2017). Malignant transformation of sinonasal inverted papilloma and related genetic alterations: A systematic review. Eur. Arch. Oto-Rhino-Laryngol..

[36] Yang Z., Zhang Y., Wang X., Huang J., Guo W., Wei P., Li G., Wang Z., Huang Z., Zhang L. (2019). Putative biomarkers of malignant transformation of sinonasal inverted papilloma into squamous cell carcinoma. J. Int. Med. Res..

[37] Hoffman Y., Bublik D.R., Pilpel Y., Oren M. (2014). miR-661 downregulates both Mdm2 and Mdm4 to activate p53. Cell Death Differ..

[38] Ryu S.W., Jeong H.J., Choi M., Karbowski M., Choi C. (2010). Optic atrophy 3 as a protein of the mitochondrial outer membrane induces mitochondrial fragmentation. Cell Mol. Life Sci..

[39] Bausch D., Thomas S., Mino-Kenudson M., Fernández-del C.C., Bauer T.W., Williams M., Warshaw A.L., Thayer S.P., Kelly K.A. (2011). Plectin-1 as a novel biomarker for pancreatic cancer. Clin. Cancer Res..

[40] Puiffe M.L., Le Page C., Filali-Mouhim A., Zietarska M., Ouellet V., Tonin P.N., Chevrette M., Provencher D.M., Mes-Masson A.M. (2007). Characterization of ovarian cancer ascites on cell invasion, proliferation, spheroid formation, and gene expression in an in vitro model of epithelial ovarian cancer. Neoplasia.

[41] Bartel D.P. (2009). MicroRNAs: Target recognition and regulatory functions. Cell.

[42] Kakizaki T., Hatakeyama H., Nakamaru Y., Takagi D., Mizumachi T., Sakashita T., Kano S., Homma A., Fukuda S. (2017). Role of microRNA-296-3p in the malignant transformation of sinonasal inverted papilloma. Oncol. Lett..

[43] Stambolic V., MacPherson D., Sas D., Lin Y., Snow B., Jang Y., Benchimol S., Mak T.W. (2001). Regulation of PTEN transcription by p53. Mol. Cell.

[44] da Costa A.A., D’Almeida Costa F., Ribeiro A.R., Guimarães A.P., Chinen L.T., Lopes C.A., de Lima V.C. (2015). Low PTEN expression is associated with worse overall survival in head and neck squamous cell carcinoma patients treated with chemotherapy and cetuximab. Int. J. Clin. Oncol..

[45] Yasukawa S., Kano S., Hatakeyama H., Nakamaru Y., Takagi D., Mizumachi T., Suzuki M., Suzuki T., Nakazono A., Tanaka S. (2018). Genetic mutation analysis of the malignant transformation of sinonasal inverted papilloma by targeted amplicon sequencing. Int. J. Clin. Oncol..

[46] Maisch S., Mueller S.K., Traxdorf M., Weyerer V., Stoehr R., Iro H., Hartmann A., Agaimy A. (2020). Sinonasal papillomas: A single centre experience on 137 cases with emphasis on malignant transformation and EGFR/KRAS status in “carcinoma ex papilloma”. Ann. Diagn. Pathol..

[47] Uchi R., Jiromaru R., Yasumatsu R., Yamamoto H., Hongo T., Manako T., Sato K., Hashimoto K., Wakasaki T., Matsuo M. (2021). Genomic Sequencing of Cancer-related Genes in Sinonasal Squamous Cell Carcinoma and Coexisting Inverted Papilloma. Anticancer Res..

[48] Wang H., Li H., Hu L., Wang J., Liu Q., Wang D., Sun X. (2019). Overexpression of FoxM1 in Sinonasal Inverted Papilloma and Associated Squamous Cell Carcinoma. Am. J. Rhinol. Allergy.

[49] Cabal V.N., Menendez M., Vivanco B., Potes-Ares S., Riobello C., Suarez-Fernandez L., Garcia-Marin R., Blanco-Lorenzo V., Lopez F., Alvarez-Marcos C. (2020). EGFR mutation and HPV infection in sinonasal inverted papilloma and squamous cell carcinoma. Rhinology.

[50] Outzen K.E., Grøntveld A., Jørgensen K., Clausen P.P., Ladefoged C. (1996). Inverted papilloma: Incidence and late results of surgical treatment. Rhinology.

[51] Mortuaire G., Arzul E., Darras J.A., Chevalier D. (2007). Surgical management of sinonasal inverted papillomas through endoscopic approach. Eur. Arch. Oto-Rhino-Laryngol..

[52] Busquets J.M., Hwang P.H. (2006). Endoscopic resection of sinonasal inverted papilloma: A meta-analysis. Otolaryngol.-Head Neck Surg..

[53] Peng R., Thamboo A., Choby G., Ma Y., Zhou B., Hwang P.H. (2019). Outcomes of sinonasal inverted papilloma resection by surgical approach: An updated systematic review and meta-analysis. Int. Forum Allergy Rhinol..

[54] Goudakos J.K., Blioskas S., Nikolaou A., Vlachtsis K., Karkos P., Markou K.D. (2018). Endoscopic Resection of Sinonasal Inverted Papilloma: Systematic Review and Meta-Analysis. Am. J. Rhinol. Allergy.

[55] Woodworth B.A., Bhargave G.A., Palmer J.N., Chiu A.G., Cohen N.A., Lanza D.C., Bolger W.E., Kennedy D.W. (2007). Clinical outcomes of endoscopic and endoscopic-assisted resection of inverted papillomas: A 15-year experience. Am. J. Rhinol..

[56] Lee T.J., Huang S.F., Huang C.C. (2004). Tailored endoscopic surgery for the treatment of sinonasal inverted papilloma. Head Neck.

[57] Miglani A., Hoxworth J.M., Zarka M.A., Lal D. (2018). Use of intraoperative negative margins reduces inverted papilloma recurrence. Am. J. Rhinol. Allergy.

[58] Banks C.A., Palmer J.N., Chiu A.G., O’Malley B.W., Woodworth B.A., Kennedy D.W. (2009). Endoscopic closure of CSF rhinorrhea: 193 cases over 21 years. Otolaryngol.-Head Neck Surg..

[59] Tong C.C.L., Patel N.N., Maina I.W., Triantafillou V., Yan C.H., Kuan E.C., Kohanski M.A., Papagiannopoulos P., Workman A.D., Cohen N.A. (2019). Inverted papilloma with multifocal attachment is associated with increased recurrence. Int. Forum Allergy Rhinol..

[60] Vrabec D.P. (1975). The inverted Schneiderian papilloma: A clinical and pathological study. Laryngoscope.

[61] Lee J.T., Yoo F., Wang M., Vengerovich G., Suh J.D. (2020). Modified endoscopic Denker approach in management of inverted papilloma of the anterior maxillary sinus. Int. Forum Allergy Rhinol..

[62] Maina I.W., Tong C.C.L., Baranov E., Patel N.N., Triantafillou V., Kuan E.C., Kohanski M.A., Papagiannopoulos P., Yan C.H., Workman A.D. (2019). Clinical Implications of Carcinoma In Situ in Sinonasal Inverted Papilloma. Otolaryngol. Head Neck Surg..

[63] Healy D.Y., Chhabra N., Metson R., Holbrook E.H., Gray S.T. (2016). Surgical risk factors for recurrence of inverted papilloma. Laryngoscope.

[64] Li W., Lu H., Zhang H., Sun X., Hu L., Wang D. (2020). Squamous cell carcinoma associated with inverted papilloma: Recurrence and prognostic factors. Oncol. Lett..

[65] Yan C.H., Newman J.G., Kennedy D.W., Palmer J.N., Adappa N.D. (2017). Clinical outcomes of sinonasal squamous cell carcinomas based on tumor etiology. Int. Forum Allergy Rhinol..

[66] Kim D.Y., Hong S.L., Lee C.H., Jin H.R., Kang J.M., Lee B.J., Moon I.J., Chung S.K., Rha K.S., Cho S.H. (2012). Inverted papilloma of the nasal cavity and paranasal sinuses: A Korean multicenter study. Laryngoscope.

[67] Yu H.X., Liu G. (2014). Malignant transformation of sinonasal inverted papilloma: A retrospective analysis of 32 cases. Oncol. Lett..

[68] Kuan E.C., Frederick J.W., Palma Diaz M.F., Lim D.W., Suh J.D. (2017). Complete response of skull base inverted papilloma to chemotherapy: Case report. Allergy Rhinol. (Provid. R.I.).

[69] Adriaensen G.F., Lim K.H., Georgalas C., Reinartz S.M., Fokkens W.J. (2016). Challenges in the Management of Inverted Papilloma: A Review of 72 Revision Cases. Laryngoscope.

